# Percutaneous Transtracheal Jet Ventilation with Various Upper Airway Obstruction

**DOI:** 10.1155/2015/454807

**Published:** 2015-06-16

**Authors:** Tomoki Doi, Tetsuya Miyashita, Ryousuke Furuya, Hitoshi Sato, Shunsuke Takaki, Takahisa Goto

**Affiliations:** Department of Anesthesiology, Yokohama City University Hospital, 3-9 Fukuura Kanazawa, Yokohama, Kanagawa 236-0004, Japan

## Abstract

A “cannot-ventilate, cannot-intubate” situation is critical. In difficult airway management, transtracheal jet ventilation (TTJV) has been recommended as an invasive procedure, but specialized equipment is required. However, the influence of upper airway resistance (UAR) during TTJV has not been clarified. The aim of this study was to compare TTJV using a manual jet ventilator (MJV) and the oxygen flush device of the anesthetic machine (AM). We made a model lung offering variable UAR by adjustment of tracheal tube size that can ventilate through a 14-G cannula. We measured side flow due to the Venturi effect during TTJV, inspired tidal volume (TVi), and expiratory time under various inspiratory times. No Venturi effect was detected during TTJV with either device. With the MJV, TVi tended to increase in proportion to UAR. With AM, significant variations in TVi was not detected with changes in any UAR. In conclusion, UAR influenced forward flow of TTJV in the model lung. The influence of choked flow from the Venturi effect was minimal under all UAR settings with the MJV, but the AM could not deliver sufficient flow.

## 1. Introduction

An unexpected difficult airway (cannot-ventilate, cannot-intubate status; CVCI) represents a critical situation requiring immediate attention. In 2004, the Difficult Airway Society guidelines recommended percutaneous transtracheal jet ventilation (TTJV) following cannula cricothyroidotomy, as an invasive procedure to address incomplete upper airway obstruction [1, 2]. This procedure is easier and quicker than surgical cricothyroidotomy [3, 4]. Manual jet ventilators (MJVs) are commonly used for TTJV via a 14-G cannula.

However, cannula cricothyroidotomy may be associated with major problems in which insufficient oxygen may be inspired by the patient via the cannula cricothyroidotomy. One of the problems is that the inspired oxygen fraction (FIO_2_) of TTJV may not be 1.0, because two different flows (the main jet flow, FIO_2_ = 1.0, and side flow due to the Venturi effect, FIO_2_ = 0.21) contribute to the actual FIO_2_ [5]. Another, more serious problem is that sufficient tidal volume may not be obtained by TTJV due to the loss of inspired flow to the upper airway, which may depend on upper airway resistance (UAR).

Furthermore, several investigators have reported contradictory findings that the oxygen flush device of anesthesia machines (AMs) should or should not be used as a substitute for MJVs [6–9]. Since the MJV is a highly specialized and uncommon device, we also tried using the oxygen flush device attached to the AM for TTJV and compared this to the MJV in a lung model with variable UAR.

To investigate oxygenation and ventilation during TTJV, using an MJV or AM, we measured side flow due to the Venturi effect during TTJV in Study 1 and inspired tidal volume (TVi) and expiratory time under various inspiratory times and UARs during TTJV in Study 2.

## 2. Methods

**Figure 1:**
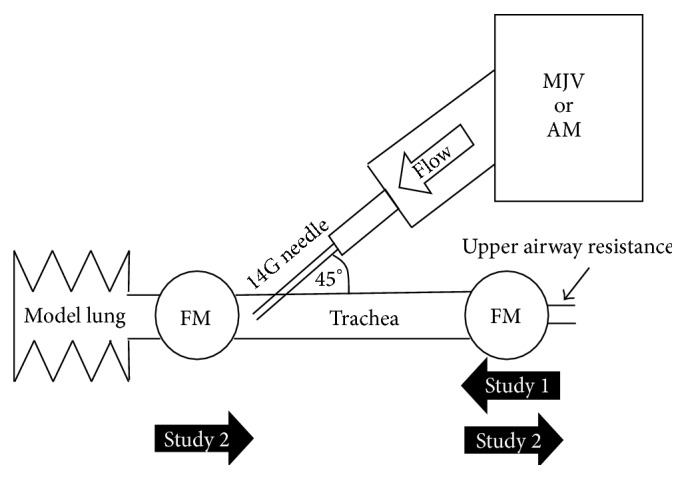
Schematic of the experimental model. MJV, manual jet ventilator (MCS-3, Yutaka, Japan); AM, anesthetic machine (Aisys, GE Healthcare, USA); FM, flowmeter. The “Study 1” arrow indicates the direction of Venturi effect flow during oxygen flush. The “Study 2” arrow indicates the direction of flow during oxygen flush.

A schematic of our experimental model is shown in Figure 1. An MJV (MCS-3; Yutaka, Japan) and an AM (Aisys, GE Healthcare, USA) with a relief valve (over 1 psi) were prepared in this study. The MJV was connected to the central oxygen port (0.35 MhPa = 50 psi). Release flow volumes with different oxygen pressures and cannula sizes for the MJV, according to the manufacturer's manual, are shown in Table 1. Oxygen release from the MJV was 1250 mL/s at the settings used in this study. A lung model (Lung Simulator; SMS Technologies, Essex, UK) was used in this study. The compliance value of the model lung was adjusted to 50 mL/cmH_2_O. The circuit of the AM or the MJV was connected to the model trachea via a 14-G (1.75-inch) catheter for the cannula cricothyroidotomy. A 14-gauge catheter (Jelco; Smith Medical, MN, USA; 1.75 inches) was placed into the simulated trachea at an angle of 45° toward the model lung. This angle of 45° is commonly recommended [10]. Two kinds of flowmeter were used in this study: one to measure the speed of flow in Study 1 and the other (Haloscale Standard; nSpire Health, CO, USA) to measure the volume of flow in Study 2.

### 2.1. Study 1

To measure flow due to the Venturi effect, we created a simulated trachea model (Figure 1). A flowmeter (Certifier FA Test System, TSI, MN, USA) was placed on the oral side of the simulated trachea to measure inhaled flow. The MJV (50 psi) or AM (45 psi) with relief valve closed was connected to the 14-gauge catheter. We measured choked flow due to the Venturi effect during the oxygen flush for 1 s with or without the connection to the model lung.

### 2.2. Study 2

UAR was adjusted between 2.5 and 6.0 mm internal diameter (ID) of the endotracheal tubes (Portex; Smith Medical, MN, USA) adjusted 10 mm of the length on the oral side of the simulated trachea (UAR of 6.0 mm ID simulated no obstruction, 2.5 mm ID simulated severe obstruction). Inspiratory time was 1 s using the MJV or 1, 2, or 3 s using the AM. We used 2 flowmeters, on the oral and lung sides of the cannula, to measure loss of tidal volume (LTV) on the oral-side airway and expiratory tidal volume (TVe) obtained from expiratory flow of the model lung during oxygen flush. Simultaneously, expiratory time with each UAR using the MJV or AM was measured on a stopwatch. Calculated minute volume (cMV) was determined from the following formula: TVe × 60/(inspiratory time + expiratory time). TVe was determined as excellent if >500 mL; good if >100 mL but ≤500 mL; fair if >50 mL but ≤100 mL; or not acceptable if ≤50 mL and MV was determined as excellent if >5.0 L/min; good if >2.0 L/min but ≤5.0 L/min; fair if >0.5 L/min but ≤2.0 L/min; or not acceptable if ≤0.5 L/min.

### 2.3. Statistical Analyses

Each measurement was repeated 6 times in this study. Data are expressed as mean ± standard deviation. Analysis of variance and Tukey's test as a post hoc test were used for statistical analyses. Values of *P* < 0.05 were considered statistically significant.

## 3. Results

### 3.1. Study 1

**Figure 2:**
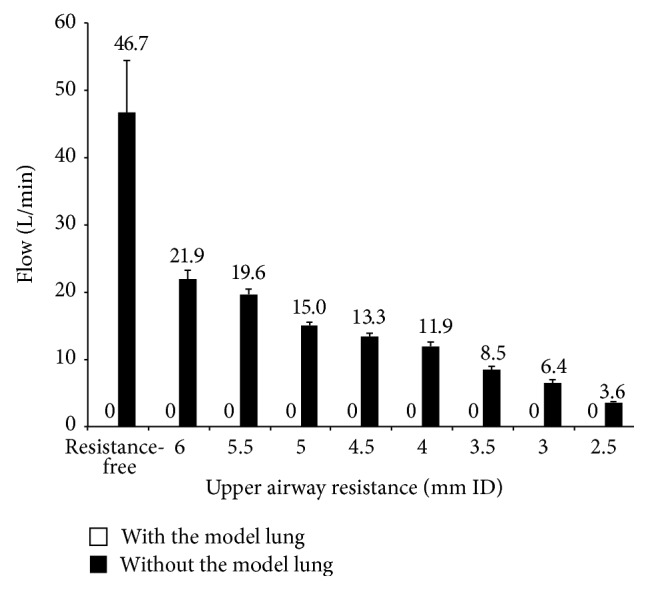
Relationship between UAR and Venturi effect with the MJV. Venturi flows were shown by connection with or without the model lung. Numeric data represents mean values. No flows were detected with the model lung.

The data from Study 1 are shown in Figure 2. With the MJV, although choked flow due to the Venturi effect was detected in each setting without connection to the model lung, no choked flow was detected on connection to the model lung. With the AM, no choked flow was detected in any setting, with or without connection to the model lung.

### 3.2. Study 2

**Figure 3:**
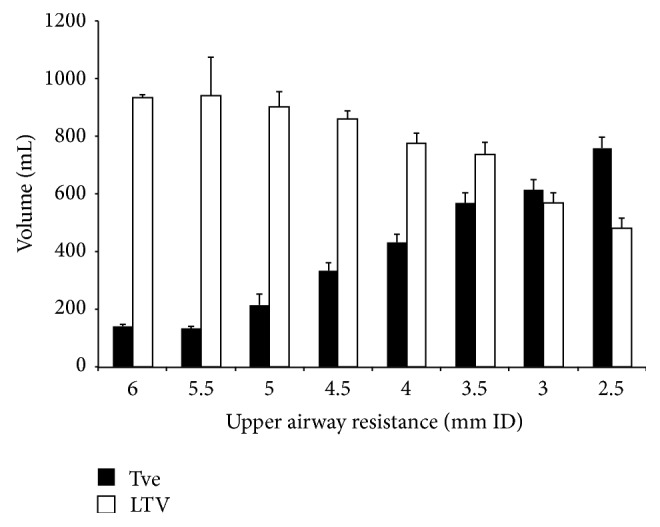
Relationship between upper airway resistance and expiratory tidal volume or loss of tidal volume. Significant differences in expiratory tidal volume were seen with upper airway resistance, except between 3.5 mm ID and 3 mm ID. TVe, expiratory tidal volume; LTV, loss of tidal volume.

**Figure 4:**
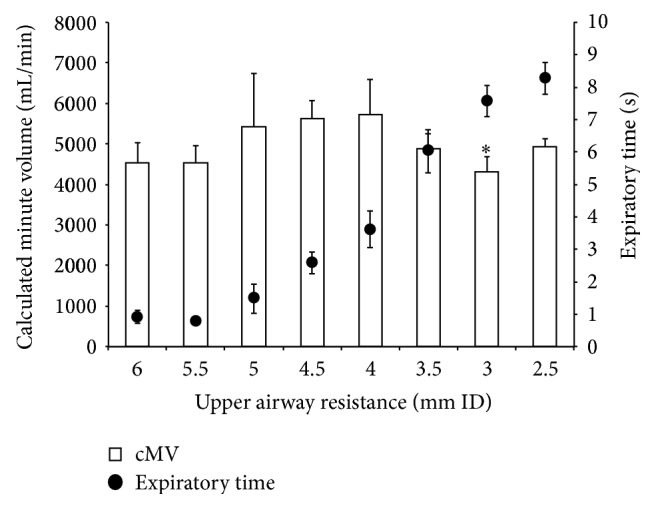
Relationship between upper airway resistance and calculated minute volume or expiratory time. A significant difference in calculated minute volume (*P* = 0.47) was apparent between 3 mm ID and 4 mm ID of upper airway resistance. cMV, calculated minute volume.

Increased UAR was associated with increased TVe, decreased LTV, and increased expiratory time with the MJV (Figure 3). Excellent tidal volumes were obtained for UAR <3.5 mm ID with the MJV. However, even with >4.0 mm ID, good tidal volumes were obtained. Increased UAR was associated with prolonged expiratory time. Peak cMV with the MJV was obtained at a UAR of 4.0 mm ID (Figure 4). With the AM, no tidal volume or minute volume was obtained with UAR >3.0 mm ID. At a UAR of 2.5 mm ID with the AM, tidal volume and minute volume were detected but were not acceptable (TVe: 1 s flush, 16.7 ± 5.1 mL; 2 s flush, 14.2 ± 4.9 mL; 3 s flush, 38.3 ± 7.5 mL).

## 4. Discussion

When the trachea was connected to the model lung, FIO_2_ during TTJV was determined as almost 1.0, because choked flow due to the Venturi effect was not detected in our simulated model in this study. The catheter angle of 45° to the trachea and/or lung compliance might have reduced flow due to the Venturi effect. Actually, it was clinically impossible to place a catheter parallel to the trachea. Also, although lung compliance of 50 mL/cmH_2_O was slightly lower than normal, we thought that it might suitably reflect compliance due to lung edema in situations of CVCI [11–15].

UAR might influence oxygen delivery to the model lung more strongly during TTJV in this study. However, excellent or good minute volume was seen with all UAR settings with the MJV, because when UAR is lower, tidal volume is lower and expiratory time is shorter. In this study, tidal volume of approximately 140 mL and expiratory time of approximately 1 s, resulting in a calculated minute volume of 4.5 L/min, were obtained with UAR at 6.0 mm ID. Tidal volume of approximately 760 mL and expiratory time of approximately 8 s, which resulted in a calculated minute volume of approximately 4.0 L/min, were obtained with UAR at 2.5 mm ID. Maximum minute volume was obtained with oral-side obstruction of 4.0 mm ID. With severe upper airway obstruction < 3.5 mm ID representing the situation of CVCI, TTJV with the MJV can sufficiently inflate the lung, but expiratory time was increased and minute volume decreased with moderate upper airway obstruction. When lung compliance is lower than 50 mL/cmH_2_O, peak minute volume must be obtained with a UAR less than 4.0 m ID because of the decreased tidal volume and shortened expiratory time.

A previous study found that AMs without relief valves can be used for TTJV [8, 9]. However, no acceptable tidal volume was obtained using a modern AM in the present study. Although the driving pressure for the oxygen flush was almost 45 psi with the AM, the limit of the relief valves in recent AMs has ranged between approximate 1 and 5 psi [16, 17]. The oxygen flush flow must not enter the model lung and escape to the expiratory port of the AM through the relief valve. However, with the UAR setting of 2.5 mm ID, slight tidal volume was obtained with a 3 s oxygen flush. If obstruction narrows the airway to <2.5 mm ID, acceptable tidal volume might be obtained.

Another method of oxygen delivery in cannula cricothyroidotomy is a direct connection between the cannula and tube from another oxygen supply. Fassl et al. investigated the driving pressure of sources of oxygen, such as wall-mounted oxygen flowmeters and AM auxiliary oxygen flowmeters [18]. The oxygen flush mechanisms of several kinds of AMs were found to be unable to deliver sufficient flows, as seen in the present study. They concluded that several flowmeters were acceptable to use as oxygen sources for TTJV in their study [18], but these methods needed specialized devices to connect the catheter for TTJV.

This study was limited to a simulated model with a model lung. To investigate more about the physiology of manual jet ventilation, we should repeat this study in animal models in future.

## 5. Conclusion

UAR influenced forward flow in TTJV of the model lung. The influence of choked flow from the Venturi effect was minimal in all settings of UAR with the MJV. The oxygen flush device of the AM could not deliver sufficient flow.

## Figures and Tables

**Table 1:** Oxygen release volume (mL/s) with MJV.

Adjustment pressure (psi)	14 Gauges	16 Gauges	18 Gauges	20 Gauges

20	690	340	210	130
30	970	390	260	200
40	1180	540	320	230
50	1250	650	380	280

Nippon Megacare Co.; Ltd. Web Page.

http://www.megacare.co.jp/products/02_mcs3.html Accessed by 23 Feb, 2014.

## References

[1] Henderson J. J., Popat M. T., Latto I. P., Pearce A. C. (2004). Difficult Airway Society guidelines for management of the unanticipated difficult intubation. *Anaesthesia*.

[2] Caplan R. A., Benumof J. L., Berry F. A. (2003). Practice guidelines for the management of difficult airway: an updated report by the American Society of Anesthesiologists Task Force on the Management of the Difficult Airway. *Anesthesiology*.

[3] Craven R. M., Vanner R. G. (2004). Ventilation of a model lung using various cricothyrotomy devices. *Anaesthesia*.

[4] Wong D. T., Lai K., Chung F. F., Ho R. Y. (2005). Cannot intubate-cannot vintilate and difficult intubation strategies: results of a Canadian national survey. *Anesthesia and Analgesia*.

[5] Baraka A. (1996). Oxygen enrichment of entrained room air during Venturi jet ventilation of children undergoing bronchoscopy. *Paediatric Anaesthesia*.

[6] Patel R. G. (1999). Percutaneous transtracheal jet ventilation: a safe, quick, and temporary way to provide oxygenation and ventilation when conventional methods are unsuccessful. *Chest*.

[7] Carl M. L., Rhee K. J., Schelegle E. S., Green J. F. (1994). Effects of graded upper-airway obstruction on pulmonary mechanics during transtracheal jet ventilation in dogs. *Annals of Emergency Medicine*.

[8] Gaughan S. D., Benumof J. L., Ozaki G. T. (1993). Can an anesthesia machine flush valve provide for effective jet ventilation?. *Anesthesia and Analgesia*.

[9] Scuderi P. E., McLeskey C. H., Comer P. B. (1982). Emergency percutaneous transtracheal ventilation during anesthesia using readily available equipment. *Anesthesia & Analgesia*.

[10] Mittal M. K. (2014). *Needle Cricothyroidotomy with Percutaneous Transtracheal Ventilation*.

[11] Udeshi A., Cantie S. M., Pierre E. (2010). Postobstructive pulmonary edema. *Journal of Critical Care*.

[12] van Kooy M. A., Gargiulo R. F. (2000). Postobstructive pulmonary edema. *American Family Physician*.

[13] Lang S. A., Duncan P. G., Shephard D. A. E., Ha H. C. (1990). Pulmonary oedema associated with airway obstruction. *Canadian Journal of Anaesthesia*.

[14] Moore R. L., Binger C. A. (1927). The response to respiratory resistance: a comparison of the effects produced by partial obstruction in the inspiratory and expiratory phases of respiration. *The Journal of Experimental Medicine*.

[15] Warren M. F., Peterson D. K., Drinker C. K. (1942). The effects of heightened negative pressure in the chest, together with further experiments upon anoxia in increasing the flow of lung lymph. *American Journal of Physiology*.

[16] Thompson C., Halder S., Wimbush S. (2007). Effective ventilation following emergency needle cricothyroidotomy. *Resuscitation*.

[17] Cook T. M., Nolan J. P., Magee P. T., Cranshaw J. H. (2007). Needle cricothyroidotomy. *Anaesthesia*.

[18] Fassl J., Jenny U., Nikiforov S., Murray W. B., Foster P. A. (2010). Pressures available for transtracheal jet ventilation from anesthesia machines and wall-mounted oxygen flowmeters. *Anesthesia and Analgesia*.

